# High visual acuity revealed in dogs

**DOI:** 10.1371/journal.pone.0188557

**Published:** 2017-12-05

**Authors:** Olle Lind, Ida Milton, Elin Andersson, Per Jensen, Lina S. V. Roth

**Affiliations:** 1 Cognitive Science, Lund University, Lund, Sweden; 2 IFM Biology, AVIAN Behaviour Genomics and Physiology Group, Linköping University, Linköping, Sweden; Indian Institute of Science, INDIA

## Abstract

Humans have selectively bred and used dogs over a period of thousands of years, and more recently the dog has become an important model animal for studies in ethology, cognition and genetics. These broad interests warrant careful descriptions of the senses of dogs. Still there is little known about dog vision, especially what dogs can discriminate in different light conditions. We trained and tested whippets, pugs, and a Shetland sheepdog in a two-choice discrimination set-up and show that dogs can discriminate patterns with spatial frequencies between 5.5 and 19.5 cycle per degree (cpd) in the bright light condition (43 cd m^-2^). This is a higher spatial resolution than has been previously reported although the individual variation in our tests was large. Humans tested in the same set-up reached acuities corresponding to earlier studies, ranging between 32.1 and 44.2 cpd. In the dim light condition (0.0087 cd m^-2^) the acuity of dogs ranged between 1.8 and 3.5 cpd while in humans, between 5.9 and 9.9 cpd. Thus, humans make visual discrimination of objects from roughly a threefold distance compared to dogs in both bright and dim light.

## Introduction

World-wide, around 400 pure dog breeds are recognised, all descended from wolves [1–4], and selectively bred by humans for different phenotypic traits or behaviours over many thousands of years. It is not clear how this domestication has affected the senses of dogs, but it may be assumed that many human activities that dogs participate in, and have been selected for, have served to uphold a strict demand on sensory abilities. For example, in vision, high performance in behaviours such as guarding, herding, hunting and social interactions requires acute spatial vision.

Humans and dogs have eyes adapted for vision in both bright and dim light, but the retinal specialisations are different. Humans have a highly specialized region of the central retina with densely packed cones and ganglion cells, the fovea, that mediates high visual acuity in bright light. Peripherally, the human retina has a lower ganglion cell density and is dominated by rods that accommodate dim light vision. Dogs have a more variable arrangement, where some dogs have retained the wolf characteristics of a horizontal streak that allows them to see sharply along the horizon while others have less pronounced visual streaks or an area centralis that, similar to the human fovea, allows for very sharp vision in a small portion of the visual field [5–7]. Intriguingly, it has been suggested that the visual streak is found in breeds with long skulls such as greyhounds while the area centralis is found in breeds with short skulls such as pugs [6]. Just like wolves, but different from humans, dogs have a *tapetum lucidum*–a cellular mirror that reflects unabsorbed light back onto the photoreceptors–that enhances the sensitivity of rod mediated vision in dim light [8–9]. How these physiological differences translate into difference in performance such as visual acuity in bright and dim light is not known.

Visual acuity depends on the density of photoreceptors and ganglion cells, focal length, and the quality of the optics [10]. When all of these factors are known, it is possible to predict an animal’s anatomical visual acuity, which can be compared with electrophysiological measurements of the cortex or the retina and the results from behavioural experiments. All of these methods have been used to describe visual acuity in beagles and mixed breeds, but in contrast to the robust data on humans e.g. [11], the results from these studies are ambiguous, spanning from about 2 to nearly 50 cycles per degree (cpd; Table 1) [5, 12–15]. This makes it difficult to compare visual perception in dogs and humans, which would be helpful to better understand human-dog interactions. Dogs have become an important research animal due to the diversity and genetic composition and there is an urgent need to reveal the visual capabilities to be able to design future behavioural experiments [16]. Here, we aim to improve our knowledge about dog visual acuity and how it relates to human performance. We trained and tested dogs and humans both in bright and dim light conditions using a two-choice discrimination set-up.

**Table 1:** Estimates of visual acuity in dogs.

Breed	Sample size	Acuity (cpd)	Light intensity	Method
Mixed breed	1	4–6.2	0.1–37 (lux)	Behaviour [15]
Mixed breed/ Beagle	4	11.6–12.6	86 (cd m^-2^)	Electro-physiology [14]
Beagle	4	2.5–4.3	86 (cd m^-2^)	Electro-physiology [13]
Beagle	3	7.1–9.5	73 (cd m^-2^)	Electro-physiology [12]
Mixed breed	18	25–46	-	Anatomical [5]

Sample size is for individuals except for the last row where sample size denotes the number of eyes from which cone densities were calculated and used to calculate an anatomical acuity estimate.

## Material and methods

### Stimuli and light intensities

**Fig 1:**
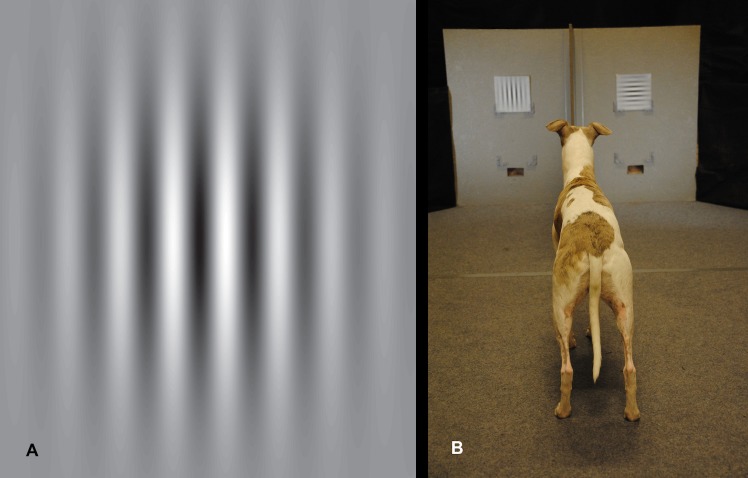
**Stimulus example (A) and the two-choice discrimination set-up (B)**. The separator forced the dog to choose between the two stimuli at a distance of 1.5 m.

The stimuli were constructed in matlab (v R2014a; all scripts are available on request), where a sinusoidal matrix was filtered by a Gaussian function (standard deviation 0.22) to create “Gabor patches” that eliminate possible edge effects e.g. [17]. These were saved as eps-images and adjusted for size in Adobe Illustrator (CS4; v 14.0.0). The images were printed (Canon image press C7010VP) on 20 x 20 cm large white paper cards (300 g multidesign paper). The resulting stimuli appeared as black and white gratings with maximal contrast at the centre (81% Michelson contrast), and decreasing contrast towards the periphery (Fig 1A).

The dogs were trained to associate a card with either horizontal (half of the subjects) or vertical gratings with food rewards. In the tests, the dogs were tested for their ability to discriminate between one card displaying horizontal, and one card displaying vertical gratings. The visual acuity threshold was determined from repeating these tests while increasing the spatial frequency (stripe width and separation) of the gratings (rewarded and non-rewarded in unison) from trial to trial.

In the bright light condition, we used stimuli with spatial frequencies of 2–30 cycles per degree (cpd) for the dogs and 30–78 cpd for humans. In the dim light condition, we used spatial frequencies of 0.5–10 cpd for dogs and 0.5–14 cpd for humans. Hence, we performed tests in two light intensities; 43 cd m^-2^ and 0.0087 cd m^-2^ (hereafter called the “bright” and the “dim” condition), as measured from an angle of 90° from a white card at the position of the stimuli. These conditions were produced by fluorescent tube in the ceiling (PHILIPS. Master TL5 HE 28W/830) and LED spots on each side of the set-up (4000K, Star trading) with neutral density filters (0.6 Neutral density filters, 210 Lee filter) directed towards the stimuli. A spectroradiometer (ILT950, International Light) was used to measure the intensities and make sure all stimulus positions received the approximately same amount of light (coefficient of variation = 0.043).

### Experimental set-up

Two grating stimuli were presented on a grey wall (1.44 m wide) on each side of a separator (Fig 1B). The stimuli could be placed either low (0.26 m above ground) or high (0.56 m above ground) depending on the dog’s preference. Below the lowest option the reward (Frolic ®) could be provided through trapdoors. There was a food reward behind each door which were manually operated by the experimenter via attached strings from behind the dog and owner. The owner who was instructed to look down released the dog 2–3 m away from the stimuli. At a distance of 1.5 m the dogs were forced to make their choice due to the separator and the angular resolution of the stimuli was calculated from this distance. At 1.5 m, there were also marks on the carpet and when one paw passed the marks, the experimenter either opened the trapdoor to release the reward if the choice was correct or let the door remain closed if the choice was wrong.

In the bright light condition, human subjects made their choice at a distance of 4 m to match the higher visual resolution in humans with higher spatial frequencies of the stimulus gratings. In the dim light condition humans made their choice at 1.5 m just like the dogs. Each stimulus subtended a visual angle of 2.9° and 7.6° at a distance of 4 m and 1.5 m respectively. The same gratings were used for both light intensities and both for dogs and humans.

### Animals and human subjects

All dogs included in the study were privately owned and recruited through personal contacts, social media or via radio announcement. Humans were recruited through personal contacts. The experiments in this study comply with the Swedish and European regulation for the use of animal and human subjects for research purposes. This means that all dog owners and human subjects were informed about the study and gave their written consent that they voluntarily participated in the study which was executed in Linköping, Sweden between May 2015-January 2016. Since we only used privately owned dogs that were only trained to associate stimuli with a reward and since the human subjects were only asked to note the orientation of stimuli gratings ethical permission was not needed (S1 Text).

Dog owners were given instructions to train the dogs in their home (May-August 2015) by displaying the stimulus together with the food reward over approximately 20 repetitions twice a week in different rooms and environments, and to introduce the command “choose” when releasing the dog. In the first 100 repetitions, only the correct stimulus was used and the dog was rewarded immediately when it looked at the correct stimulus. In next stage, an alternative stimulus (again, horizontal or vertical stripes printed on paper) was shown to the dog at the same time as the correct one. When the owner introduced both stimuli they were instructed to ignore incorrect choices. They were asked also to present the correct stimulus approximately equal number of times to the left and to the right and to never have three consecutive correct presentations on the same side. We also encouraged the owners to present the stimuli at different heights above the floor to see whether that improved the dogs’ performance.

In August, all dogs started formal training in the experimental set-up in the bright light condition (43 cd m^-2^) and the dogs reaching the learning criterion (15/20 correct choices at two consecutive training occasions) were allowed to continue to the tests. This resulted in the inclusion of four whippets and three pugs rewarded for horizontal patterns, and one Shetland sheepdog rewarded for vertical patterns. All included dogs were examined by a veterinarian with indirect ophthalmoscopy and slit lamp to assess the status of their eyes (Table 2). After completion of data collection in daylight we started to train the dogs in dim light (0.0087 cd m^-2^). The learning criterion in dim light was six consecutive correct choices and four whippets and three pugs reached this criterion but due to an accident (unrelated to the experiment) the Shetland sheepdog could not continue with the testing in dim light. Human subjects (five females, three males; Table 3) were tested in the same set-up as the dogs, but were not trained before data collection.

**Table 2:** Veterinary eye examination report for the four whippets, three pugs and one Shetland sheepdog included in the study.

Name	Breed	Sex	Age	Eye examination by veterinarian
Sniff	whippet	Male	11 years	Partial equatorial cataract in lens cortex, expected to have no/little impact on vision
Acke	whippet	Male	6 years	Within normal variation
Gaia	whippet	Female	4 years	Within normal variation
Dafne	whippet	Female	5 years	Within normal variation
Poppe	pug	Male	3 years	Tiny pigmentation of the cornea expected to have no/little impact on vision
Bosse*	pug	Male	3 years	Heavily pigmented cornea on both eyes (50–75%) expected to have large impact on vision
Doris*	pug	Female	10 months	Tiny cloudy dot in cornea expected to have no/little impact on vision
Pesto	Shetland sheepdog	Male	4 years	Within normal variation

*Excluded from final analysis because of poor performance in tests (see text).

**Table 3:** Eye status, age and sex of the eight humans (F: females; M: males) included in the study.

Name	Sex	Age (years)	Eyeglasses/contact lenses
F1	Female	33	Yes (short-sighted)
F2	Female	24	No
F3	Female	35	No (slight astigmatism)
F4	Female	23	Yes (long-sighted)
F5*	Female	24	No
M1	Male	23	No
M2	Male	29	No
M3	Male	49	No

*Excluded from final analysis because of poor performance in tests (see text).

### Training and test sessions

One session contained 15–40 trials which was adjusted depending on the endurance of the dog and after discussions with the dog owner. For some dogs a short break was necessary to maintain high motivation. During training sessions in the bright light condition only frequencies of 1 and 3 cpd were used and these were pseudo-randomized, both with respect to side for the correct pattern and for the order of stimulus frequency presentation. However, the correct stimulus could be positioned a maximum of two consecutive times at the same side.

During test sessions, i.e. after the learning criterion for bright light had been reached, the frequencies 2, 4, 6, 8, 10, 12, 14 and 16 cpd were included in a pseudo-random manner. Again, correct stimulus was presented equally often to the left and right. For some dogs, additional frequencies (18, 20, 22, 24, 26, 28 and 30 cpd) were added due to high number of correct choices. However, in all these predetermined schedules the first trial was always stimulus of low frequency. In addition, before each test session (when data was collected) the dog was presented with two warm-up trials not included in data collection to make sure they remembered the task. Only the 20 first choices for each frequency were used for all dogs and the complete data set can be found in Table C in S1 File.

Humans performed 20 choices of each of the frequencies 30, 39, 44, 50, 64, 70 and 78 cpd. However, for humans, one frequency at the time was tested (i.e. 20 trials with 30 cpd followed by 20 trials of 39 cpd and so on), starting with the lowest frequency but again the position of the correct stimulus was pseudo-randomized.

Before each session in dim light, all dogs and humans were allowed to fully adapt to darkness for 30 min [9,18]. After fulfilling the learning criterion for dim light, dogs were tested as in the bright light condition but for grating frequencies of 0.5, 2, 4, 6, 8 and 10 cpd, and for humans, we included also 12 and 14 cpd.

### Data analyses

For each individual, we used a maximal likelihood procedure executed by a modified version of the free matlab program Palamedes (v. 1.6.2) [19], to fit a Weibull function to the experimental data describing correct choice frequency as a function of spatial frequency of the grating stimulus.

$$\psi (x)=\gamma +(1-\gamma -\lambda )\left(1-e^{-{\left(\frac{x}{a}\right)}^{b}}\right),$$ (1)

where *ψ* is the correct choice frequency at stimulus intensity *x*, *γ* is the guess rate, the lower asymptote of the function (fixed to 0.5), λ is the lapse rate (the difference between the upper asymptote and 1, allowed to vary between 0 and 0.2), and *a* and *b* are unrestricted parameters representing slope position and steepness respectively. For the fitted psychometric functions, we interpolated the threshold at 75% correct choice (which represents a significant bias at the p < 0.05 level in a one-tailed binomial distribution, n = 20).

### Subjects included in analysis

The eyes of the dogs included in the final analysis were examined by a veterinarian (A. Weidman), who concluded that the minor abnormalities observed (Table 2) were assumed to have little or no expected impact of vision. One of the pugs (Bosse) that was later excluded from the analysis due to low number of correct choices had heavily pigmented eyes which could have had a large negative impact on his vision according to the veterinarian.

Hence, we started the experiments with eight dogs; four whippets, three pugs, and one Shetland sheepdog. However, two of the pugs (Doris and Bosse) performed poorly in the experiments with a maximal correct choice frequency of 77%. These dogs were excluded from further analysis, as we could not fit the psychometric function to the experimental data. The results from the Shetland sheepdog (Pesto) are incomplete, as we did not test this dog in dim light (see Animals and human subjects and Table A-B in S1 File). Of eight humans starting the experiments, one female (F5) was excluded from further analysis, as she only scored 60% correct choices even at the lowest stimulus frequency level.

## Results

**Fig 2:**
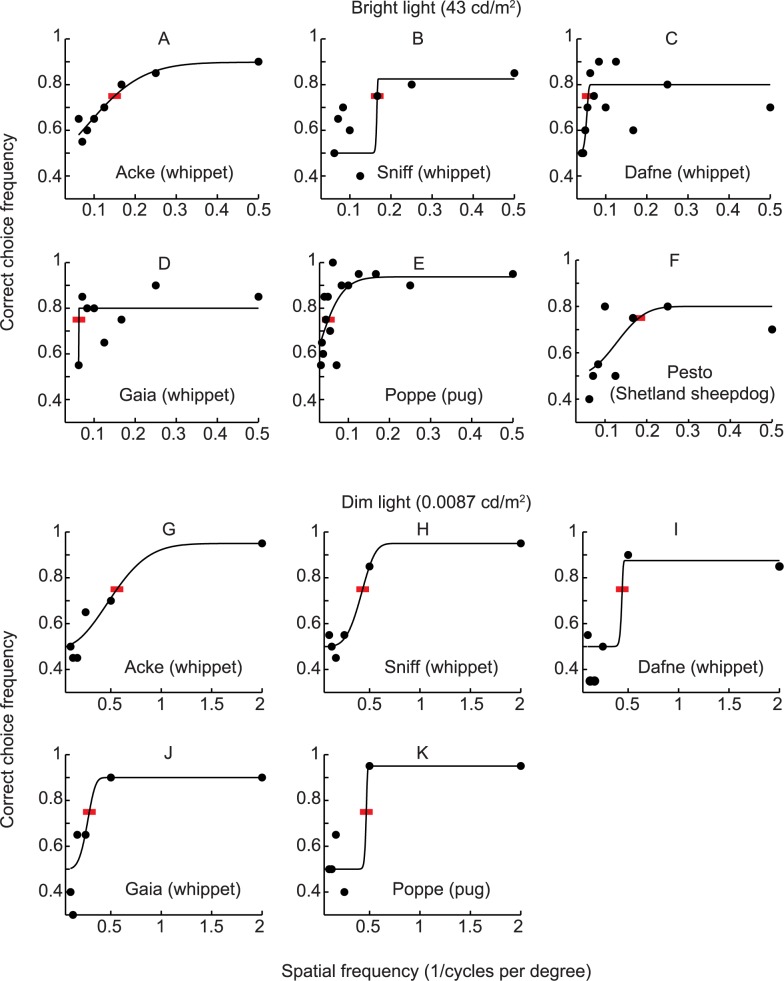
**Visual acuity of four whippets (A-D, G-J), one pug (E, K), and one Shetland sheepdog (F) in bright and dim light conditions.** Lines represent the psychometric functions fitted to the experimental data (filled circles, each representing 20 choices) and red bars indicate where the acuity thresholds were interpolated.

**Fig 3:**
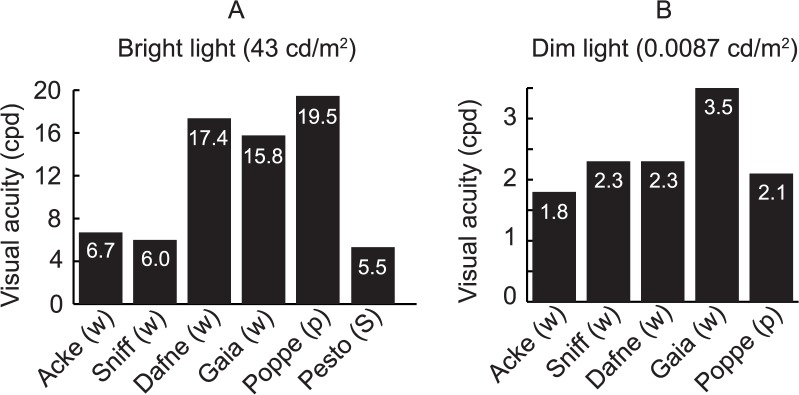
Bar diagrams clarifying visual acuity. **Visual acuity** was acquired from interpolating thresholds at 75% correct choices (see Fig 2). Breed abbreviation within brackets.

The visual acuity of the dogs ranged between 5.5 and 19.5 cpd in bright light (Figs 2A–2F and 3A), and between 1.8 and 3.5 cpd in the dim light condition (Figs 2G–2L and 3B). Performance robustness, measured as the upper asymptote of the fitted psychometric function, was lower in the bright (ranging between 80–89% correct choices) compared to the dim test condition (ranging between 87.5–95% correct choices; Fig 2). The variability of the behavioural data was large, therefore we also calculated thresholds based on the highest frequency with a significantly biased choice frequency (see Table A in S1 File and S1 Fig). This did not affect our general conclusions, although some changes in the results for individuals were observed (as an increase in maximal acuity from 19.5 cpd, to 24 cpd for the pug named Poppe; see Table A in S1 File and S1 Fig). The S1 and S2 Movies show two of the dogs discriminating between horizontal and vertical gratings in the two-choice discrimination test.

**Fig 4:**
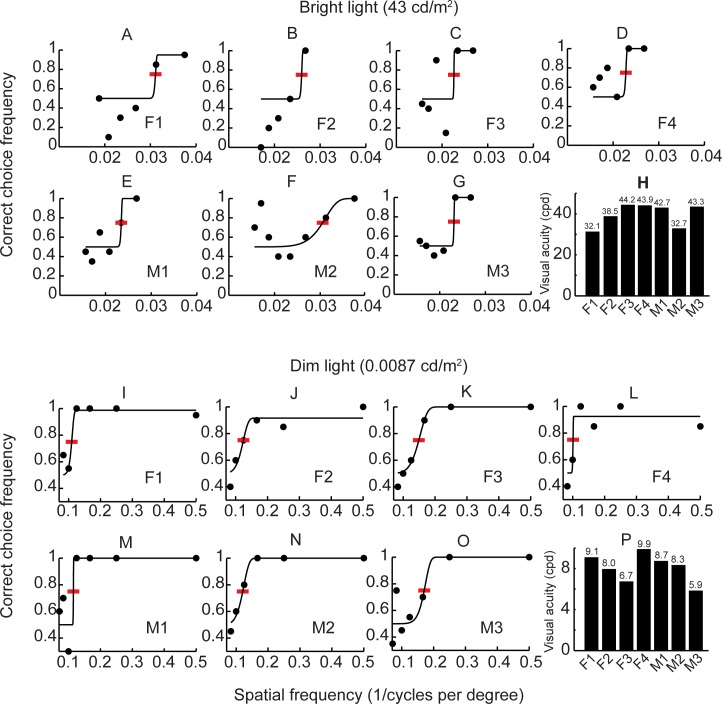
**Visual acuity of seven humans in bright (A-H) and dim (I-P) light conditions.** Lines represent the psychometric functions fitted to the experimental data (filled circles) and red bars indicate where the acuity thresholds were interpolated. The inserts in the bar diagrams clarify visual acuity that was acquired from interpolating thresholds at 75% correct choices.

In humans, visual acuity ranged between 32.1 and 44.2 cpd in the bright light condition (Fig 4A–4H), and between 5.9 and 9.9 cpd in the dim light condition (Fig 4I–4P). In contrast to the dogs, performance robustness did not change between the two light conditions.

## Discussion

**Fig 5:**
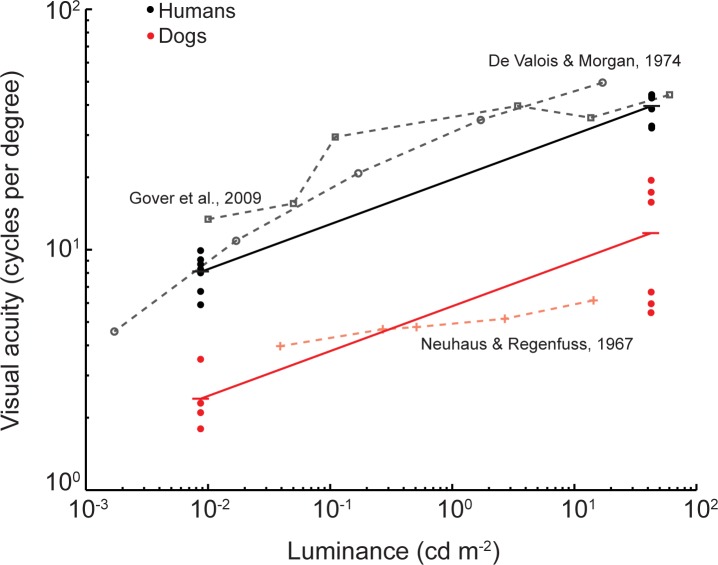
Visual acuity as a function of stimulus intensity in dogs (red) and humans (black, grey). Present data are shown as filled circles for each individual, and averages are shown as dashes connected by solid lines. Data from literature are shown as grey circles and squares for humans, and plus signs for dogs connected by dashed lines. The data from Neuhaus and Regenfuss [15] was originally reported in lux units and is roughly translated into the units of cd m^-2^ by taking the lighting conditions and experimental setup into account. Lines connecting our results for individual subjects in bright and dim light have been omitted for clarity. We could not find any correlation between the acuity rankings in bright and dim light for dogs or humans (Figs 3 and 4).

Our study revealed a higher visual acuity in dogs in bright light conditions than what would be expected from earlier studies. While the visual acuity of on Shetland sheepdog and two of the whippets matched the results from earlier behavioural studies on dogs (5.5–6.2 cpd) [15], the acuity of the other two whippets and the pug was two to four times higher (Fig 3 and Table 1). Roughly, on average, the visual acuity of humans is three times higher than in dogs in both bright and dim light conditions (Fig 5).

Anatomical estimates based upon the density of cone photoreceptors and focal length suggests that visual acuity in dogs can be in the range of 25 to 46 cpd [5]. These calculations are likely overestimations, as they do not account for the convergence of cones onto ganglion cells, neither are they corrected for optical aberrations. Ganglion cell density may be controlled by only a few genes [7] and indeed, ganglion cell density is shown to vary largely both between breeds [6], within breeds, and even within the same litter [7]. This could at least partly explain the differences between anatomical and experimental results, and the high variation we observed in the present data.

The large variation between individuals was surprising and also that a pug reached higher acuities (19.5 cpd) than the whippets (6.7–15.8 cpd), which are sight hounds bred for speed and excellence of hunting by sight [20]. On the other hand, the pug is being bred as a companion dog [21], resulting in frontally placed eyes which might have higher visual acuity enabling the dog to see small changes in facial expressions in their owner. McGreevy and colleagues [6] suggested that short skulled (brachycephalic) breeds such as the pug, have an area centralis with very high ganglion cell density that allow for higher visual acuity in a small limited region of the visual field compared to the visual streak of long skulled breeds (dolichocephalic) such as the whippet. The horizontal streak in wolves is thought to help them to keep track of pack members during hunting while keeping the gaze direction on the prey [22]. This could possibly also be the case in sighthounds. However, it might also be that temporal vision, to see fast movement, is more important than high spatial acuity [20] but this needs further investigations. With the small number of dogs in our study we cannot confirm nor can we reject any hypothesis about breed differences. Furthermore, the difference between the area centralis and the visual streak may motivate investigations of how the orientation of the rewarded pattern has an effect on the achieved visual acuity threshold.

Our results on visual acuity in humans are in agreements with earlier studies (Fig 5), despite the rather poor fits of the psychometric functions to the data (Fig 4A–4G). The surprisingly low choice frequencies by some of the human subjects (Fig 4A and 4B) are troubling and difficult to explain. We scrutinized the testing procedures but found no errors in the protocols or the reports. Still, we cannot exclude that human errors caused the results. Alternatively, some human subjects experienced aliasing of the stimulus gratings, which can appear in many different forms and cause confusion during discrimination [11]. Following our data, we suggest that humans have a visual acuity that is roughly three times higher than that of dogs in both bright and dim light conditions (Fig 5). Thus, in order to distinguish some visual detail that humans just can perceive, dogs need to reduce the distance to the object by a factor of three.

We found that the dogs’ performance was more robust in the dim light condition than in the bright light condition (i.e. around 90% correct choices in dim light and 80% in bright light; Fig 2). We do not believe that the dogs improved performance from learning (dim light experiments started after the bright light experiments for all dogs) as we could not detect any learning effects when scrutinizing the result sequences. Possibly, the dogs were more concentrated on the task in dim light, possibly from less distraction from the surrounding environment, but such speculations cannot be resolved within the scope of this study.

Only a few of the dogs initially trained for the experiments, completed all tests. It was surprisingly difficult to make the dogs associate striped pattern with a reward during the initial training phase. This difficulty might be the reason why so few studies have been published before ours. It could have to do with an inherent difficulty in stimulus-reward association with this type of stimuli, as similar problems were described over 100 years ago [23]. Variation in training success is often found in studies on visual abilities in dogs [24] However, using real objects as stimuli and performing the visual task in the dog’s home environment could be a more fruitful approach [25], but, with the different illumination conditions and the need for exact control of the stimuli, this would have been difficult to implement in our experiment.

In conclusion, in this study, we demonstrate the highest visual acuity reported from behavioural studies or electrophysiological measurements in dogs, together with large variations between breeds and individuals. It is important to acknowledge this when designing future behavioural methods since the dog is an increasingly important model animal and differences in visual ability could affect the outcome of various behavioural studies [16].

## Supporting information

S1 File Individual data. (A) First 20 choices used in analysis for the dogs. (B) Human data used in the analysis. (C) All dog data in bright light condition.
(XLSX)

S1 Fig The visual acuity thresholds of all dogs included in the study. The acuity in bright and dim (striped bars) light condition based on the highest frequency with a significantly biased choice frequency. X indicates no significant results.
(EPS)

S1 Movie Two-choice discrimination test where the whippet discriminated between horizontal and vertical gratings. (MP4)

S2 Movie Two-choice discrimination test where the Shetland sheepdog discriminated between horizontal and vertical gratings. (MP4)

S1 Text Ethical statement. (DOCX)
