# Supplementary material for: High visual acuity revealed in dogs
Source: PLoS One. 2017 Dec 5;12(12):e0188557. doi: 10.1371/journal.pone.0188557 (PMC5716585; doi:10.1371/journal.pone.0188557)
Supplement: S1 Text — (DOCX) [file pone.0188557.s005.docx]

**Ethical Statement
Animals and human subjects**

All dogs included in the study were privately owned and recruited through personal contacts, social media or via radio announcement. Humans were recruited through personal contacts. The experiments in this study comply with the Swedish and European regulation for the use of animal and human subjects for research purposes. This means that all dog owners and human subjects were informed about the study and gave their written consent that they voluntarily participated in the study which was executed in Linköping, Sweden between May 2015-January 2016. Since we only used privately owned dogs that were only trained to associate stimuli with a reward and since the human subjects were only asked to note the orientation of stimuli gratings ethical permission was not needed.
